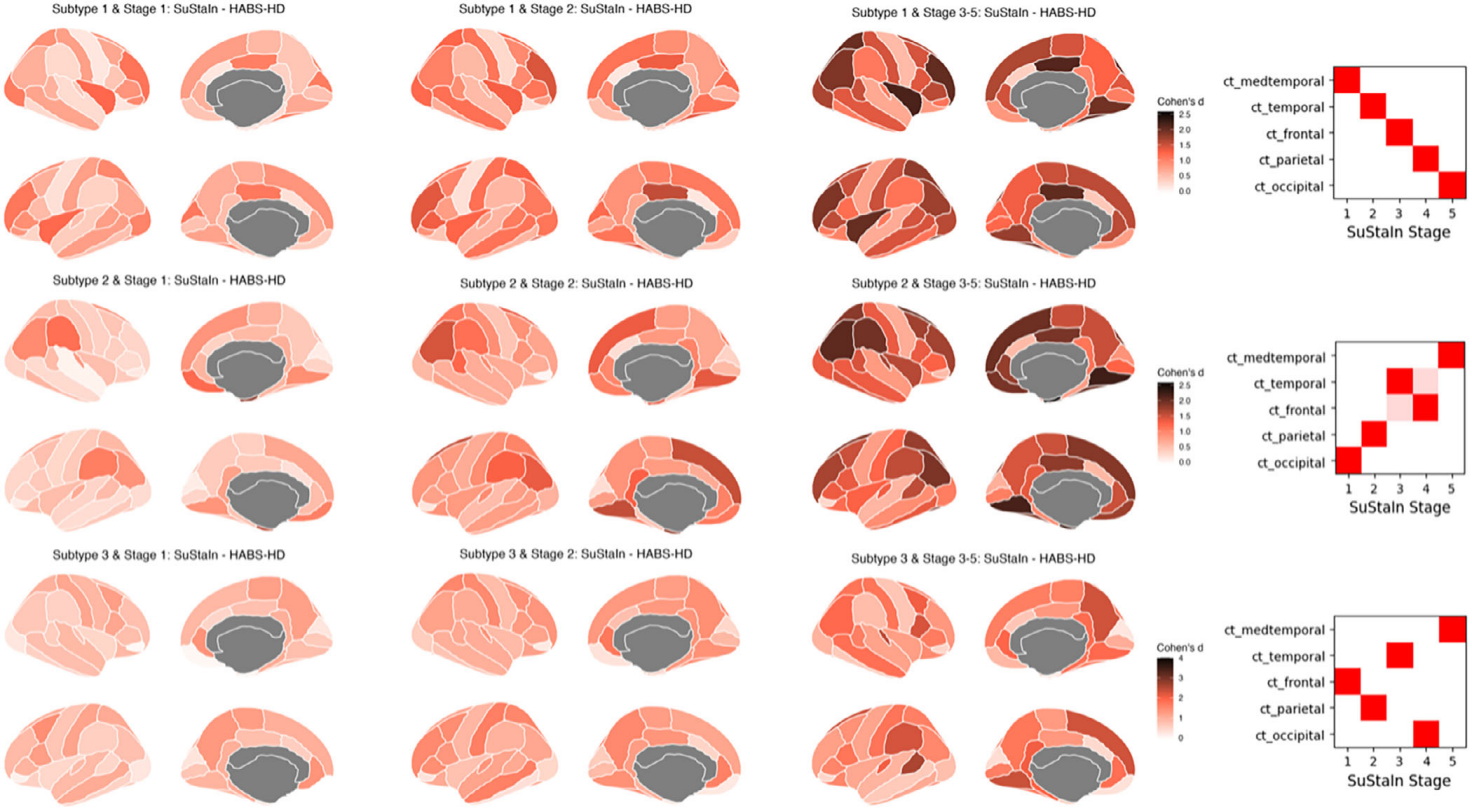# Spatial atrophy subtypes are associated with specific sociodemographic, comorbidity, pathologic, and cognitive factors in a representative community‐based cohort: a HABS‐HD study

**DOI:** 10.1002/alz70856_103619

**Published:** 2025-12-24

**Authors:** Karin L. Meeker, Gordon An, Aristeidis Sotiras, Ann D Cohen, Meredith N. Braskie, Arthur W. Toga, Sid E. O'Bryant, Beau Ances, Brian A. Gordon

**Affiliations:** ^1^ Institute for Translational Research, University of North Texas Health Science Center, Fort Worth, TX, USA; ^2^ Washington University in St. Louis, St. Louis, MO, USA; ^3^ Mallinckrodt Institute of Radiology, Washington University School of Medicine in St Louis, St Louis, MO, USA; ^4^ University of Pittsburgh School of Medicine, Pittsburgh, PA, USA; ^5^ Imaging Genetics Center, Mark and Mary Stevens Neuroimaging and Informatics Institute, Keck School of Medicine, University of Southern California, Marina del Rey, CA, USA; ^6^ Mark and Mary Stevens Neuroimaging and Informatics Institute, Keck School of Medicine, University of Southern California, Los Angeles, CA, USA; ^7^ Washington University in St. Louis School of Medicine, St. Louis, MO, USA

## Abstract

**Background:**

Biological heterogeneity is associated with heterogeneity in clinical outcomes and response to treatment in Alzheimer's disease (AD). Spatial atrophy subtypes are consistently observed across studies, although the generalizability of prior work is unknown due to limited variability within clinic‐based cohorts. The present study characterized spatial atrophy subtypes and their associated sociodemographic, comorbidity, cognitive, and pathological profiles in a large representative sample.

**Method:**

Data were obtained from the community‐based Health and Aging Brain Study – Health Disparities (HABS‐HD) and included 1,007 Hispanics, 594 Blacks, and 1,014 non‐Hispanic whites. The Subtype and Stage Inference (SuStaIn) algorithm was applied to structural MRI to derive spatial subtypes. Sociodemographic, comorbidity, cognitive, and pathologic (amyloid and tau PET, white matter hyperintensities) factors were compared by subtype.

**Result:**

In addition to individuals with minimal atrophy (Controls, *n* = 2,032), three subtypes were identified including medial‐temporal (MTL)‐predominant (Subtype 1, *n* = 299), MTL‐sparing (Subtype 2, *n* = 190), and diffuse atrophy (Subtype 3, *n* = 94). Compared to Controls, when collapsing across stage and subtype, individuals with atrophy were more likely to be Hispanic or Black, male, have less education, lower incomes, more pathology, more comorbidities, poorer cognitive performance, and more likely to be cognitively impaired (*p*'s <0.05). When assessing differences across the subtypes, MTL‐sparing individuals had greater amyloid and tau burden, poorer cognitive performance, and were more likely to be cognitively impaired compared to the other subtypes (*p*'s <0.05). The proportion of individuals in each of the subtypes was similar across ethno‐racial groups (*p*'s >0.05).

**Conclusion:**

Although a greater proportion of diverse groups demonstrate atrophy, spatial subtypes are generalizable to the general population and are associated with distinct pathologic and cognitive profiles. Clinical outcomes and response to treatment may vary by atrophy subtype and can inform clinical trials and precision‐medicine efforts.